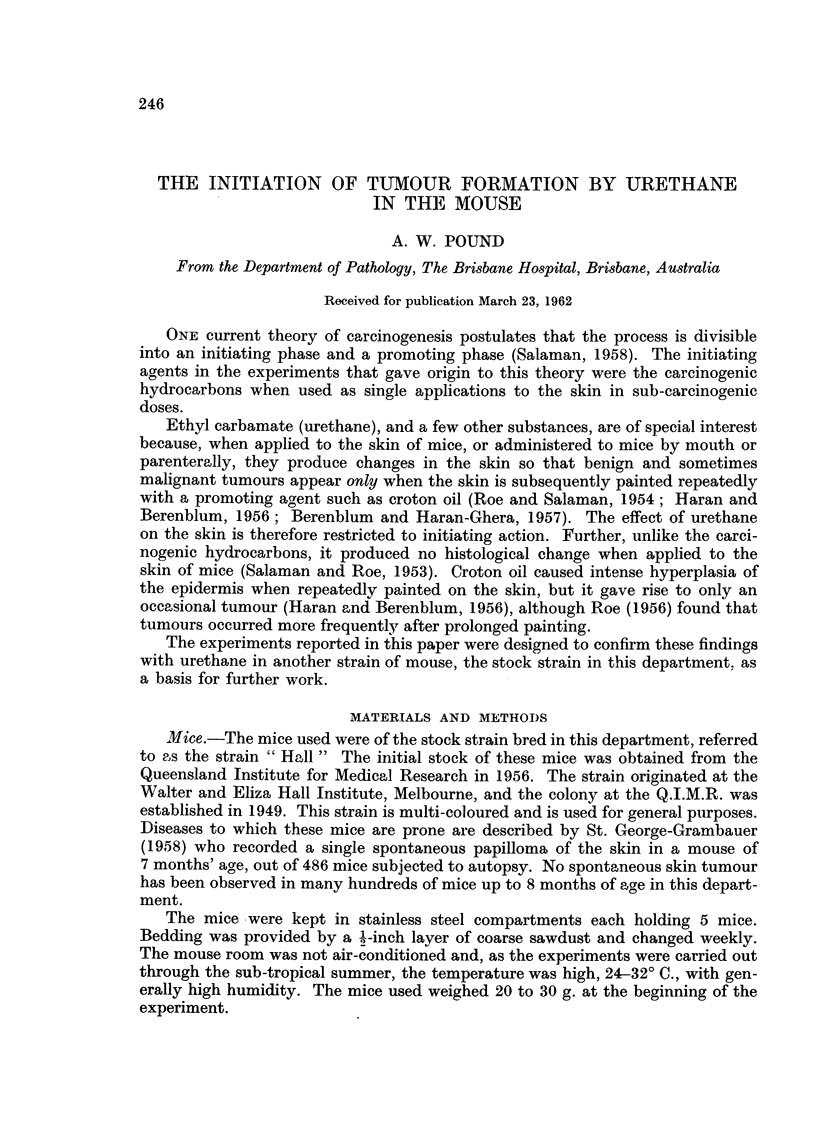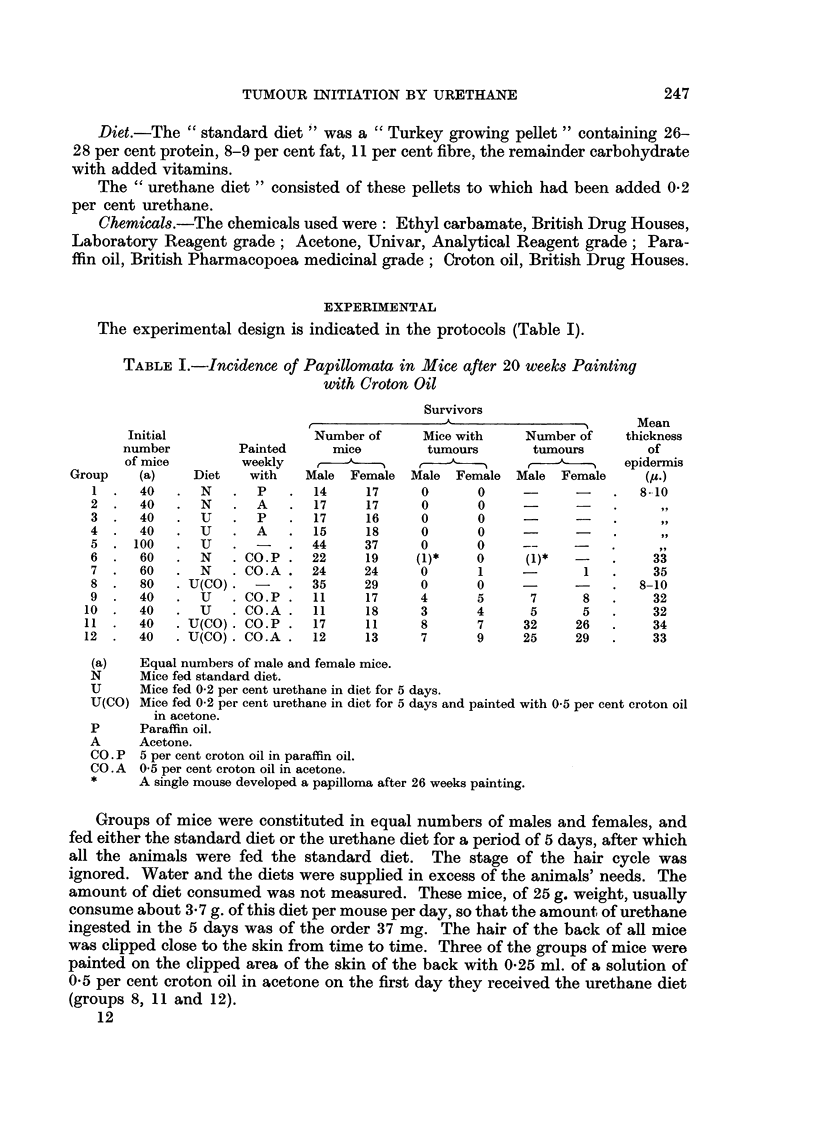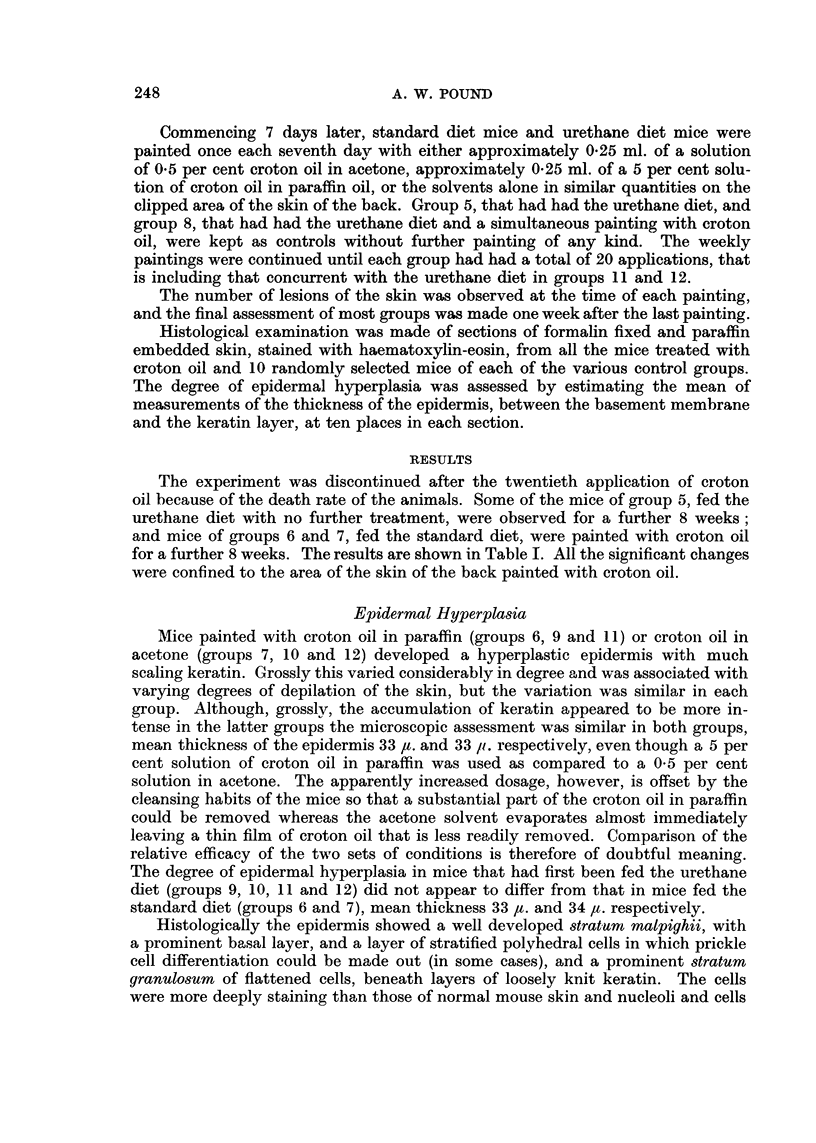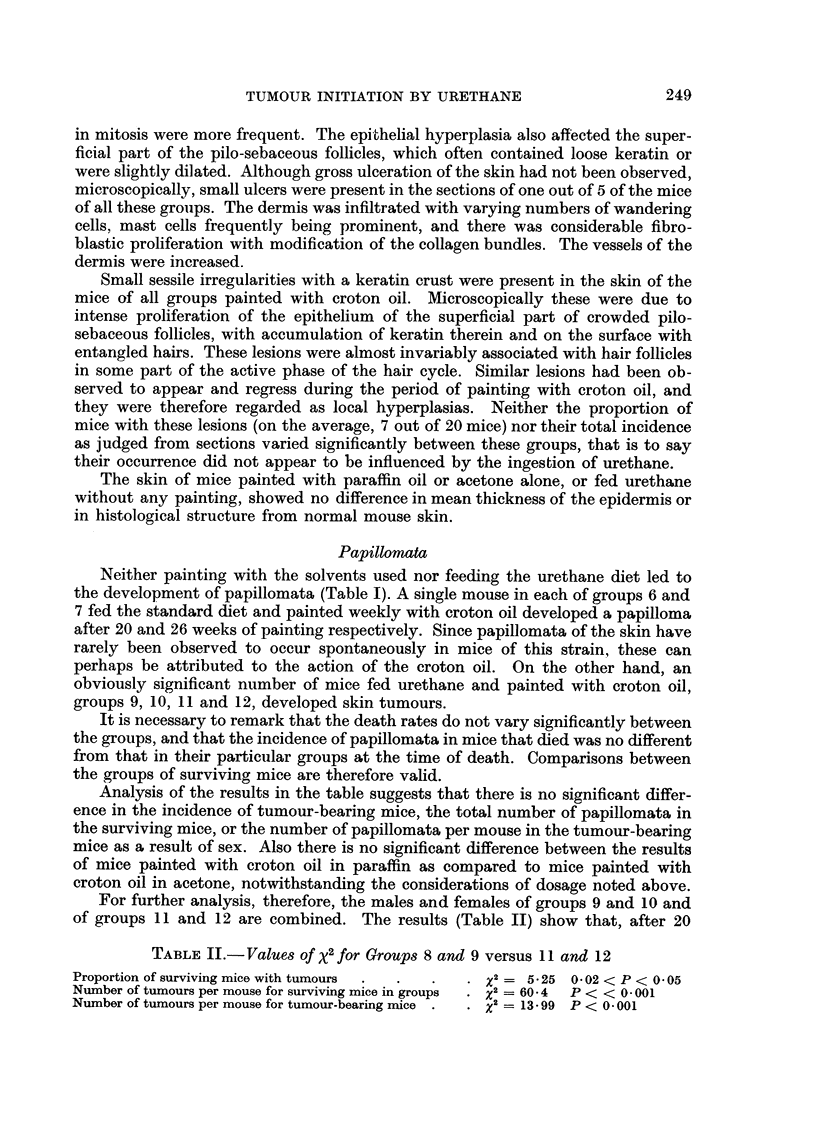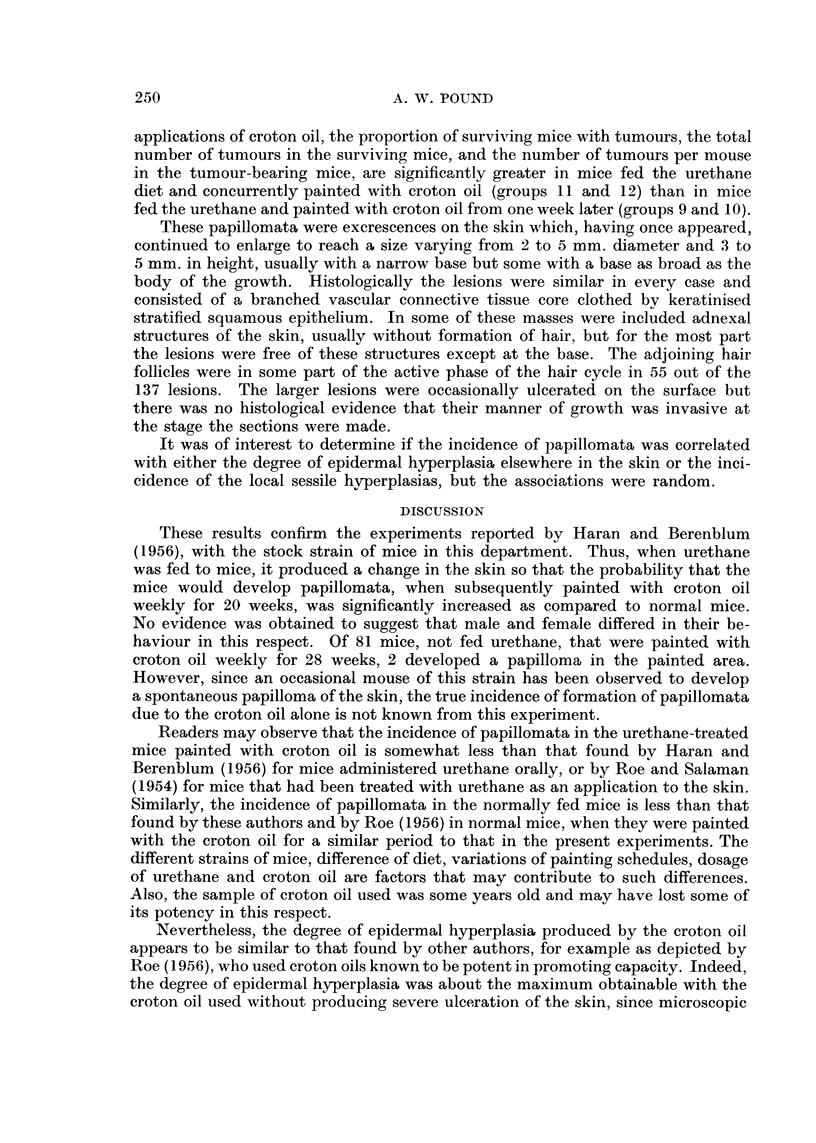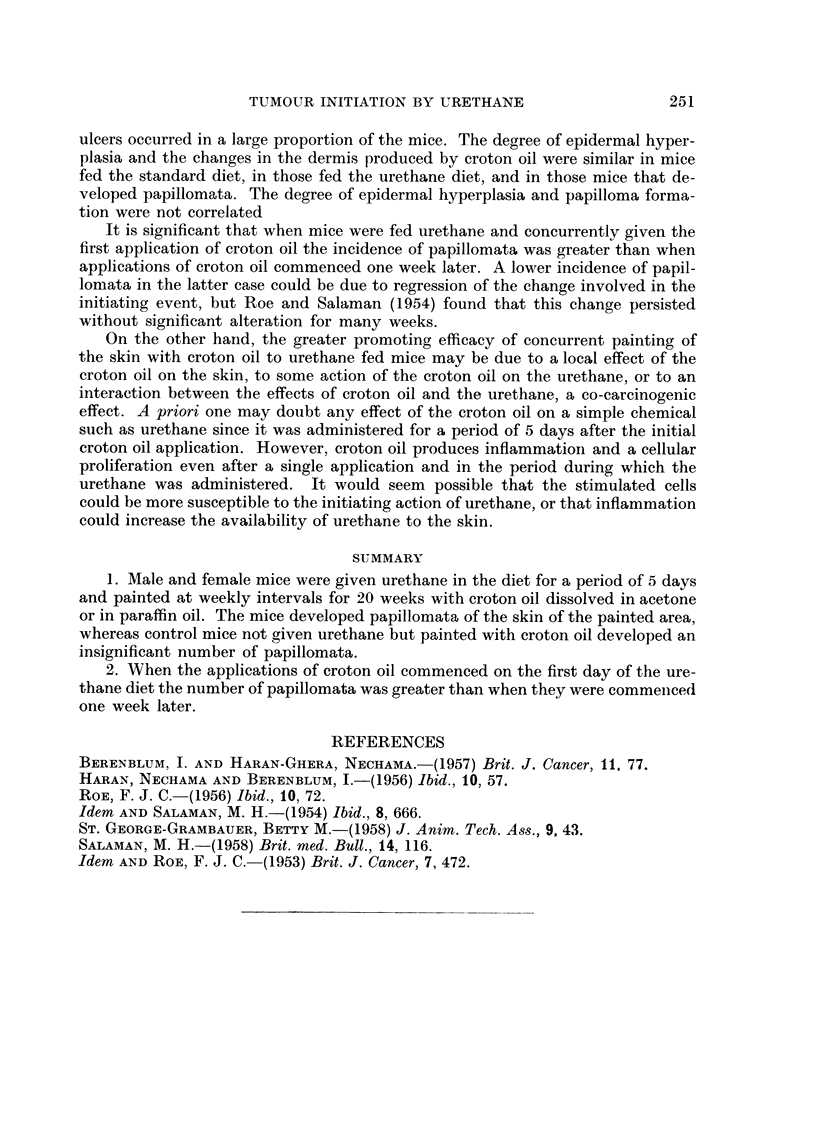# The Initiation of Tumour Formation by Urethane in the Mouse

**DOI:** 10.1038/bjc.1962.28

**Published:** 1962-06

**Authors:** A. W. Pound


### THE INITIATION OF TUMOUR FORMATION BY URETHANE IN THE MOUSE

A. W. POUND

*From the Department of Pathology, The Brisbane Hospital, Brisbane, Australia*

Received for publication March 23, 1962

ONE current theory of carcinogenesis postulates that the process is divisible into an initiating phase and a promoting phase (Salaman, 1958). The initiating agents in the experiments that gave origin to this theory were the carcinogenic hydrocarbons when used as single applications to the skin in sub-carcinogenic doses.

Ethyl carbamate (urethane), and a few other substances, are of special interest because, when applied to the skin of mice, or administered to mice by mouth or parenterally, they produce changes in the skin so that benign and sometimes malignant tumours appear *only* when the skin is subsequently painted repeatedly with a promoting agent such as croton oil (Roe and Salaman, 1954 ; Haran and Berenblum, 1956 ; Berenblum and Haran-Ghera, 1957). The effect of urethane on the skin is therefore restricted to initiating action. Further, unlike the carcinogenic hydrocarbons, it produced no histological change when applied to the skin of mice (Salaman and Roe, 1953). Croton oil caused intense hyperplasia of the epidermis when repeatedly painted on the skin, but it gave rise to only an occasional tumour (Haran and Berenblum, 1956), although Roe (1956) found that tumours occurred more frequently after prolonged painting.

The experiments reported in this paper were designed to confirm these findings with urethane in another strain of mouse, the stock strain in this department, as a basis for further work.

### MATERIALS AND METHODS

*Mice.*—The mice used were of the stock strain bred in this department, referred to as the strain " Hall " The initial stock of these mice was obtained from the Queensland Institute for Medical Research in 1956. The strain originated at the Walter and Eliza Hall Institute, Melbourne, and the colony at the Q.I.M.R. was established in 1949. This strain is multi-coloured and is used for general purposes. Diseases to which these mice are prone are described by St. George-Grambauer (1958) who recorded a single spontaneous papilloma of the skin in a mouse of 7 months' age, out of 486 mice subjected to autopsy. No spontaneous skin tumour has been observed in many hundreds of mice up to 8 months of age in this department.

The mice were kept in stainless steel compartments each holding 5 mice. Bedding was provided by a $\frac{1}{2}$-inch layer of coarse sawdust and changed weekly. The mouse room was not air-conditioned and, as the experiments were carried out through the sub-tropical summer, the temperature was high, 24–32° C., with generally high humidity. The mice used weighed 20 to 30 g. at the beginning of the experiment.

*Diet.*—The " standard diet " was a " Turkey growing pellet " containing 26–28 per cent protein, 8–9 per cent fat, 11 per cent fibre, the remainder carbohydrate with added vitamins.

The " urethane diet " consisted of these pellets to which had been added 0·2 per cent urethane.

*Chemicals.*—The chemicals used were : Ethyl carbamate, British Drug Houses, Laboratory Reagent grade ; Acetone, Univar, Analytical Reagent grade ; Paraffin oil, British Pharmacopoea medicinal grade ; Croton oil, British Drug Houses.

### EXPERIMENTAL

The experimental design is indicated in the protocols (Table I).

TABLE I.—*Incidence of Papillomata in Mice after* 20 *weeks Painting with Croton Oil*

| | | | | Survivors | | | | | | |
|---|---|---|---|---|---|---|---|---|---|---|
| | | | | Number of mice | | Mice with tumours | | Number of tumours | | |
| Group | Initial number of mice (a) | Diet | Painted weekly with | Male | Female | Male | Female | Male | Female | Mean thickness of epidermis (μ.) |
| 1 | 40 | N | P | 14 | 17 | 0 | 0 | — | — | 8-10 |
| 2 | 40 | N | A | 17 | 17 | 0 | 0 | — | — | ,, |
| 3 | 40 | U | P | 17 | 16 | 0 | 0 | — | — | ,, |
| 4 | 40 | U | A | 15 | 18 | 0 | 0 | — | — | ,, |
| 5 | 100 | U | — | 44 | 37 | 0 | 0 | — | — | ,, |
| 6 | 60 | N | CO.P | 22 | 19 | (1)* | 0 | (1)* | — | 33 |
| 7 | 60 | N | CO.A | 24 | 24 | 0 | 1 | — | 1 | 35 |
| 8 | 80 | U(CO) | — | 35 | 29 | 0 | 0 | — | — | 8–10 |
| 9 | 40 | U | CO.P | 11 | 17 | 4 | 5 | 7 | 8 | 32 |
| 10 | 40 | U | CO.A | 11 | 18 | 3 | 4 | 5 | 5 | 32 |
| 11 | 40 | U(CO) | CO.P | 17 | 11 | 8 | 7 | 32 | 26 | 34 |
| 12 | 40 | U(CO) | CO.A | 12 | 13 | 7 | 9 | 25 | 29 | 33 |

(a) Equal numbers of male and female mice.
N Mice fed standard diet.
U Mice fed 0·2 per cent urethane in diet for 5 days.
U(CO) Mice fed 0·2 per cent urethane in diet for 5 days and painted with 0·5 per cent croton oil in acetone.
P Paraffin oil.
A Acetone.
CO.P 5 per cent croton oil in paraffin oil.
CO.A 0·5 per cent croton oil in acetone.
* A single mouse developed a papilloma after 26 weeks painting.

Groups of mice were constituted in equal numbers of males and females, and fed either the standard diet or the urethane diet for a period of 5 days, after which all the animals were fed the standard diet. The stage of the hair cycle was ignored. Water and the diets were supplied in excess of the animals' needs. The amount of diet consumed was not measured. These mice, of 25 g. weight, usually consume about 3·7 g. of this diet per mouse per day, so that the amount of urethane ingested in the 5 days was of the order 37 mg. The hair of the back of all mice was clipped close to the skin from time to time. Three of the groups of mice were painted on the clipped area of the skin of the back with 0·25 ml. of a solution of 0·5 per cent croton oil in acetone on the first day they received the urethane diet (groups 8, 11 and 12).

Commencing 7 days later, standard diet mice and urethane diet mice were painted once each seventh day with either approximately 0·25 ml. of a solution of 0·5 per cent croton oil in acetone, approximately 0·25 ml. of a 5 per cent solution of croton oil in paraffin oil, or the solvents alone in similar quantities on the clipped area of the skin of the back. Group 5, that had had the urethane diet, and group 8, that had had the urethane diet and a simultaneous painting with croton oil, were kept as controls without further painting of any kind. The weekly paintings were continued until each group had had a total of 20 applications, that is including that concurrent with the urethane diet in groups 11 and 12.

The number of lesions of the skin was observed at the time of each painting, and the final assessment of most groups was made one week after the last painting.

Histological examination was made of sections of formalin fixed and paraffin embedded skin, stained with haematoxylin-eosin, from all the mice treated with croton oil and 10 randomly selected mice of each of the various control groups. The degree of epidermal hyperplasia was assessed by estimating the mean of measurements of the thickness of the epidermis, between the basement membrane and the keratin layer, at ten places in each section.

### RESULTS

The experiment was discontinued after the twentieth application of croton oil because of the death rate of the animals. Some of the mice of group 5, fed the urethane diet with no further treatment, were observed for a further 8 weeks; and mice of groups 6 and 7, fed the standard diet, were painted with croton oil for a further 8 weeks. The results are shown in Table I. All the significant changes were confined to the area of the skin of the back painted with croton oil.

### *Epidermal Hyperplasia*

Mice painted with croton oil in paraffin (groups 6, 9 and 11) or croton oil in acetone (groups 7, 10 and 12) developed a hyperplastic epidermis with much scaling keratin. Grossly this varied considerably in degree and was associated with varying degrees of depilation of the skin, but the variation was similar in each group. Although, grossly, the accumulation of keratin appeared to be more intense in the latter groups the microscopic assessment was similar in both groups, mean thickness of the epidermis 33 $\mu$. and 33 $\mu$. respectively, even though a 5 per cent solution of croton oil in paraffin was used as compared to a 0·5 per cent solution in acetone. The apparently increased dosage, however, is offset by the cleansing habits of the mice so that a substantial part of the croton oil in paraffin could be removed whereas the acetone solvent evaporates almost immediately leaving a thin film of croton oil that is less readily removed. Comparison of the relative efficacy of the two sets of conditions is therefore of doubtful meaning. The degree of epidermal hyperplasia in mice that had first been fed the urethane diet (groups 9, 10, 11 and 12) did not appear to differ from that in mice fed the standard diet (groups 6 and 7), mean thickness 33 $\mu$. and 34 $\mu$. respectively.

Histologically the epidermis showed a well developed *stratum malpighii*, with a prominent basal layer, and a layer of stratified polyhedral cells in which prickle cell differentiation could be made out (in some cases), and a prominent *stratum granulosum* of flattened cells, beneath layers of loosely knit keratin. The cells were more deeply staining than those of normal mouse skin and nucleoli and cells in mitosis were more frequent. The epithelial hyperplasia also affected the superficial part of the pilo-sebaceous follicles, which often contained loose keratin or were slightly dilated. Although gross ulceration of the skin had not been observed, microscopically, small ulcers were present in the sections of one out of 5 of the mice of all these groups. The dermis was infiltrated with varying numbers of wandering cells, mast cells frequently being prominent, and there was considerable fibroblastic proliferation with modification of the collagen bundles. The vessels of the dermis were increased.

Small sessile irregularities with a keratin crust were present in the skin of the mice of all groups painted with croton oil. Microscopically these were due to intense proliferation of the epithelium of the superficial part of crowded pilo-sebaceous follicles, with accumulation of keratin therein and on the surface with entangled hairs. These lesions were almost invariably associated with hair follicles in some part of the active phase of the hair cycle. Similar lesions had been observed to appear and regress during the period of painting with croton oil, and they were therefore regarded as local hyperplasias. Neither the proportion of mice with these lesions (on the average, 7 out of 20 mice) nor their total incidence as judged from sections varied significantly between these groups, that is to say their occurrence did not appear to be influenced by the ingestion of urethane.

The skin of mice painted with paraffin oil or acetone alone, or fed urethane without any painting, showed no difference in mean thickness of the epidermis or in histological structure from normal mouse skin.

### *Papillomata*

Neither painting with the solvents used nor feeding the urethane diet led to the development of papillomata (Table I). A single mouse in each of groups 6 and 7 fed the standard diet and painted weekly with croton oil developed a papilloma after 20 and 26 weeks of painting respectively. Since papillomata of the skin have rarely been observed to occur spontaneously in mice of this strain, these can perhaps be attributed to the action of the croton oil. On the other hand, an obviously significant number of mice fed urethane and painted with croton oil, groups 9, 10, 11 and 12, developed skin tumours.

It is necessary to remark that the death rates do not vary significantly between the groups, and that the incidence of papillomata in mice that died was no different from that in their particular groups at the time of death. Comparisons between the groups of surviving mice are therefore valid.

Analysis of the results in the table suggests that there is no significant difference in the incidence of tumour-bearing mice, the total number of papillomata in the surviving mice, or the number of papillomata per mouse in the tumour-bearing mice as a result of sex. Also there is no significant difference between the results of mice painted with croton oil in paraffin as compared to mice painted with croton oil in acetone, notwithstanding the considerations of dosage noted above.

For further analysis, therefore, the males and females of groups 9 and 10 and of groups 11 and 12 are combined. The results (Table II) show that, after 20

TABLE II.—*Values of* $\chi^2$ *for Groups* 8 *and* 9 versus 11 *and* 12

| | | |
|---|---|---|
| Proportion of surviving mice with tumours | $\chi^2 = 5 \cdot 25$ | $0 \cdot 02 < P < 0 \cdot 05$ |
| Number of tumours per mouse for surviving mice in groups | $\chi^2 = 60 \cdot 4$ | $P << 0 \cdot 001$ |
| Number of tumours per mouse for tumour-bearing mice | $\chi^2 = 13 \cdot 99$ | $P < 0 \cdot 001$ |

applications of croton oil, the proportion of surviving mice with tumours, the total number of tumours in the surviving mice, and the number of tumours per mouse in the tumour-bearing mice, are significantly greater in mice fed the urethane diet and concurrently painted with croton oil (groups 11 and 12) than in mice fed the urethane and painted with croton oil from one week later (groups 9 and 10).

These papillomata were excrescences on the skin which, having once appeared, continued to enlarge to reach a size varying from 2 to 5 mm. diameter and 3 to 5 mm. in height, usually with a narrow base but some with a base as broad as the body of the growth. Histologically the lesions were similar in every case and consisted of a branched vascular connective tissue core clothed by keratinised stratified squamous epithelium. In some of these masses were included adnexal structures of the skin, usually without formation of hair, but for the most part the lesions were free of these structures except at the base. The adjoining hair follicles were in some part of the active phase of the hair cycle in 55 out of the 137 lesions. The larger lesions were occasionally ulcerated on the surface but there was no histological evidence that their manner of growth was invasive at the stage the sections were made.

It was of interest to determine if the incidence of papillomata was correlated with either the degree of epidermal hyperplasia elsewhere in the skin or the incicidence of the local sessile hyperplasias, but the associations were random.

### DISCUSSION

These results confirm the experiments reported by Haran and Berenblum (1956), with the stock strain of mice in this department. Thus, when urethane was fed to mice, it produced a change in the skin so that the probability that the mice would develop papillomata, when subsequently painted with croton oil weekly for 20 weeks, was significantly increased as compared to normal mice. No evidence was obtained to suggest that male and female differed in their behaviour in this respect. Of 81 mice, not fed urethane, that were painted with croton oil weekly for 28 weeks, 2 developed a papilloma in the painted area. However, since an occasional mouse of this strain has been observed to develop a spontaneous papilloma of the skin, the true incidence of formation of papillomata due to the croton oil alone is not known from this experiment.

Readers may observe that the incidence of papillomata in the urethane-treated mice painted with croton oil is somewhat less than that found by Haran and Berenblum (1956) for mice administered urethane orally, or by Roe and Salaman (1954) for mice that had been treated with urethane as an application to the skin. Similarly, the incidence of papillomata in the normally fed mice is less than that found by these authors and by Roe (1956) in normal mice, when they were painted with the croton oil for a similar period to that in the present experiments. The different strains of mice, difference of diet, variations of painting schedules, dosage of urethane and croton oil are factors that may contribute to such differences. Also, the sample of croton oil used was some years old and may have lost some of its potency in this respect.

Nevertheless, the degree of epidermal hyperplasia produced by the croton oil appears to be similar to that found by other authors, for example as depicted by Roe (1956), who used croton oils known to be potent in promoting capacity. Indeed, the degree of epidermal hyperplasia was about the maximum obtainable with the croton oil used without producing severe ulceration of the skin, since microscopic ulcers occurred in a large proportion of the mice. The degree of epidermal hyperplasia and the changes in the dermis produced by croton oil were similar in mice fed the standard diet, in those fed the urethane diet, and in those mice that developed papillomata. The degree of epidermal hyperplasia and papilloma formation were not correlated

It is significant that when mice were fed urethane and concurrently given the first application of croton oil the incidence of papillomata was greater than when applications of croton oil commenced one week later. A lower incidence of papillomata in the latter case could be due to regression of the change involved in the initiating event, but Roe and Salaman (1954) found that this change persisted without significant alteration for many weeks.

On the other hand, the greater promoting efficacy of concurrent painting of the skin with croton oil to urethane fed mice may be due to a local effect of the croton oil on the skin, to some action of the croton oil on the urethane, or to an interaction between the effects of croton oil and the urethane, a co-carcinogenic effect. *A priori* one may doubt any effect of the croton oil on a simple chemical such as urethane since it was administered for a period of 5 days after the initial croton oil application. However, croton oil produces inflammation and a cellular proliferation even after a single application and in the period during which the urethane was administered. It would seem possible that the stimulated cells could be more susceptible to the initiating action of urethane, or that inflammation could increase the availability of urethane to the skin.

### SUMMARY

1. Male and female mice were given urethane in the diet for a period of 5 days and painted at weekly intervals for 20 weeks with croton oil dissolved in acetone or in paraffin oil. The mice developed papillomata of the skin of the painted area, whereas control mice not given urethane but painted with croton oil developed an insignificant number of papillomata.

2. When the applications of croton oil commenced on the first day of the urethane diet the number of papillomata was greater than when they were commenced one week later.

### REFERENCES

Berenblum, I. and Haran-Ghera, Nechama.—(1957) *Brit. J. Cancer*, **11**, 77.
Haran, Nechama and Berenblum, I.—(1956) *Ibid.*, **10**, 57.
Roe, F. J. C.—(1956) *Ibid.*, **10**, 72.
*Idem* and Salaman, M. H.—(1954) *Ibid.*, **8**, 666.
St. George-Grambauer, Betty M.—(1958) *J. Anim. Tech. Ass.*, **9**, 43.
Salaman, M. H.—(1958) *Brit. med. Bull.*, **14**, 116.
*Idem* and Roe, F. J. C.—(1953) *Brit. J. Cancer*, **7**, 472.